# Small bowel adenocarcinoma in a patient with Coeliac disease: A case report

**DOI:** 10.1186/1757-1626-1-159

**Published:** 2008-09-18

**Authors:** Mauro Lombardo, Gian Marco Giorgetti

**Affiliations:** 1 Nutritional Team, "S. Eugenio" Hospital of Rome, Rome, Italy

## Abstract

Coeliac disease is a chronic inflammatory disease of the gut with increased risk of gastrointestinal malignancy. Although enteropathy T-lymphoma is the most common neoplasm in patient affected by coeliac disease, an increased frequency of small bowel carcinoma has been described. We present a case of jejunal carcinoma in a patient suffering for coeliac disease in which gastrointestinal and extraintestinal symptoms of disease developed although he was treated with a gluten-free diet.

## Introduction

Celiac disease is a chronic inflammatory disease of the gut occurring in genetically susceptible individuals in all age groups after ingestion of gluten. It affects approximately 1% of Caucasian populations [1], though it is significantly underdiagnosed. It is characterized by a flattened mucosa, villous atrophy and crypt hyperplasia in the small intestine and by the classic malabsorption syndrome (diarrhoea, steatorrhoea, and weight loss) or by minor apparently unrelated symptoms such as iron-deficiency anemia, osteopenic bone disease, amenorrhea and infertility [2]. The lack of gluten in the diet generally leads to a return to normality of the morphological changes [3]. Malignant neoplasms of the small bowel are among the rarest types of cancer, accounting for only 2% of all GI cancers. There is little information about the presentation and prognosis of these tumours, and the frequency of established risk factors. Coeliac disease carries an increased risk of gastrointestinal malignancy: the most common neoplasm in coeliacs is jejunal T-cell lymphoma but also an increased frequency of small bowel carcinoma has been described [4]. We report a case of jejunal carcinoma in a patient suffering for coeliac disease in which gastrointestinal and extraintestinal symptoms of disease developed although he was treated with a gluten-free diet.

## Case report

In 2005 a 50-year-old caucasian male referred to another hospital due to weakness, lose of weight of about 25 kilograms, dyspepsia, diarrhoea (with about 4–5 motions every day) and episodes of postprandial vomits. The patient had been diagnosed in 1990 with coeliac disease by an esophagogastroduodenoscopy (EGD) and duodenal biopsies. He had been treated with a gluten-free diet and had some clinical improvements as disappearance of diarrhoea but continued to lose weight and had an increase of episodes of postprandial vomits. Patient was then treated as affected by a psychological disease.

**Figure 1:**
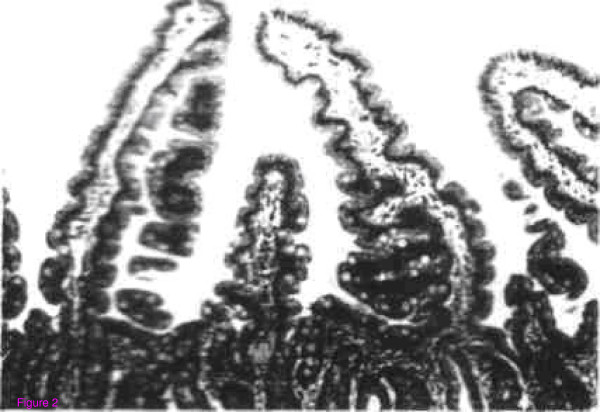
Total villous atrophy with crypt hyperplasia of the second portion of duodenum (Marsch III; lesion).

On November 2006 he was admitted to our hospital for the above mentioned symptoms. We performed an EGDS which showed an expansion of the stomach, gastric stagnation, chronic erosive gastroduodenitis, and a reduction of Kerckring's folds on the second portion of the duodenum and a mosaic pattern to the mucosa. 3 biopsy specimens are taken from the second part of the duodenum by using standard forceps, and these are submitted in formalin for analysis. Diagnosis of celiac disease was confirmed and Marsh III lesions were found (Figure 1) [5]. Patient performed a computed tomography (CT) of the superior abdomen that showed an expansion of duodenum till the ligament of Treitz and a 99 m Tc-Exametazime (HMPAO)-labeled leukocyte scintillation scanning which did not evidence any areas of progressive concentration of marked cells or any prove of phlogosis or pathology.

**Figure 2:**
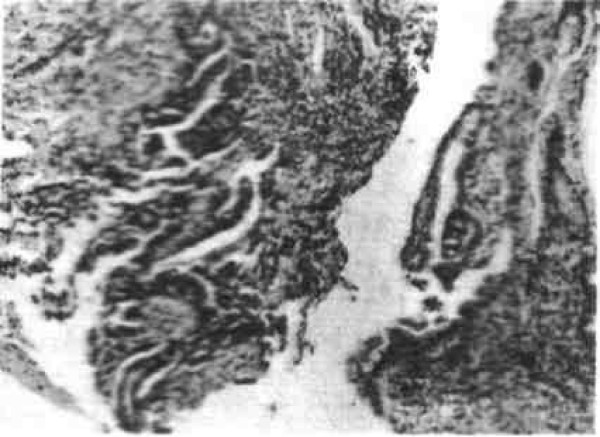
Adenocarcinoma of the proximal jejunum (40×).

**Figure 3:**
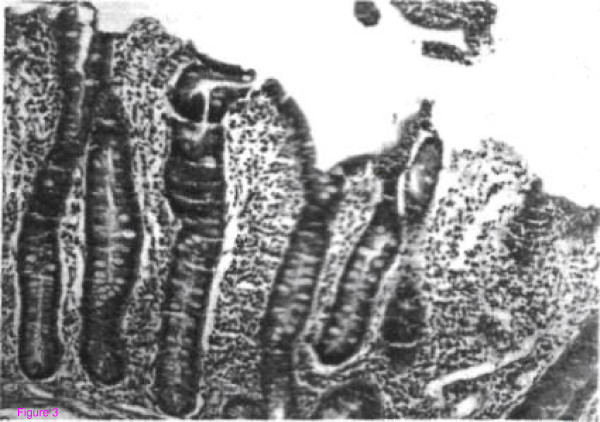
Normal histology of the small bowel 12 months after gluten withdrawal.

An explorative laparotomy showed an adenocarcinoma of the 4th portion (Figure 2) of duodenum so patient was treated surgically. Histological samples confirmed an adenocarcinoma moderately differentiated (G2) and excluded lymph nodes involvement (stage T3N0M0 AJCC VI ed. 2002). At the last examination (January 2007) he was symptom and disease free with absence of any clinical or laboratorial sign of malabsorption and negativity of serological tests of coeliac disease. Likewise, histological evaluation of small bowel mucosa showed normal villi (Figure 3) with regular size of the crypts and absence of any inflammatory infiltrate in the lamina propria (Marsh 0 according to Marsh classification). He began a parenteral nutrition for recovering weight and a better resolution of disease.

## Discussion

Celiac disease is often an underhand disease because of minor symptoms such as iron-deficiency anaemia, osteopenic bone disease, amenorrhoea and infertility. Long standing coeliac disease is associated with an increased risk of malignancy, not only of intestinal lymphoma but also small intestinal adenocarcinoma and squamous carcinoma of the oesophagus [6]. Minor symptoms such as iron-deficiency anaemia, osteopenic bone disease, amenorrhoea and infertility may be apparently unrelated to celiac disease, which is often a underhand disease.

Long standing coeliac disease is associated with an increased risk of malignancy, not only of intestinal lymphoma but also small intestinal adenocarcinoma and squamous carcinoma of the esophagus [5,6]. For these reasons some authors consider celiac disease to be a premalignant condition and suggest looking for a subclinical or silent celiac disease in all patients with diagnosis of small bowel adenocarcinoma. [7,8].

A central point in our study is that patient was symptomatic as affected of celiac disease although he was following a gluten-free diet and that these symptoms was underrated and were superficially considered as a psychological disorder [9]. This neoplasia is a significant challenge in clinical practice. Symptoms duration may range from a few weeks to several months and in most cases symptoms may occur as occlusion [10]. Symptoms should be carefully checked in coeliac patients: in patients with initial response to GFD followed by the appearance of abdominal pain, meteorism and abdominal cramps, we should exclude a partial small bowel occlusion [8]. It is very important to perform a correct diagnosis of small bowel carcinoma as soon as possible due to its poor prognosis. A recent study showed that overall actuarial 5-year survival was 38%, ranging from 0% after palliative treatment to 54% after curative resection, and the prognosis is worsened by lymph nodes and serosa involvement and if the tumour is undifferentiated.

We do not know whether an early diagnosis of coeliac disease before 1990 would prevent jejunal adenocarcinoma, since Kingham et al. recently described a case of a young female that developed small bowel adenocarcinoma recurrence fifteen years after resection of a fist small bowel carcinoma despite continuing remission of the coeliac disease [11]. In our case the patient is still symptoms-free one year after jejunal adenocarcinoma resection and in continuing remission of the coeliac disease 18 years after the start of GFD, but follow-up would be performed indefinitely, especially in light of Kingham's experience.

## Conclusion

In conclusion, this care confirms that coeliac disease is a sinister disease which may appear for the first time as severe complication such as jejunal adenocarcinoma. So, we think that screening for coeliac disease should be performed in all patients with duodenojejunal adenocarcinoma. On the other hand, it is also hypothesized that predisposition to small bowel adenocarcinoma in coeliac disease may be genetic, since only a few coeliacs develop this rare neoplasia.

## Competing interests

The authors declare that they have no competing interests.

## Authors' contributions

ML analyzed and was a major contributor in writing the manuscript. GMG performed EGDS and interpreted the patient data. All authors read and approved the final manuscript.

## Consent

Written informed consent was obtained from the patient for publication of this case report and accompanying images. A copy of the written consent is available for review by the Editor-in-Chief of this journal.

## References

[B1] Fasano A, Berti I, Gerarduzzi T (2003). Prevalence of celiac disease in at-risk and not-at-risk groups in the United States: a large multicenter study. Arch Intern Med.

[B2] Tursi A, Giorgetti GM, Brandimarte G, Rubino E, Lombardi D, Gasbarrini G (2001). Prevalence and clinical presentation of subclinical/silent coeliac disease in adults: an analysis on a 12-year observation. Hepatogastroenterology.

[B3] Rodrigo L Celiac disease. World J Gastroenterol. Review. World J Gastroenterol.

[B4] Brousse N, Meijer JW (2005). Malignant complications of coeliac disease. Best Pract Res Clin Gastroenterol.

[B5] Marsh MN (1992). Gluten, major histocompatibility complex, and the small intestine. A molecular and immunologic approach to the spectnim of gluten sensitivity (celiac sprue). Gastroenterology.

[B6] Green PH, Jabri B (2002). Celiac disease and other precursors to small-bowel malignancy. Gastroenterol Clin North Am.

[B7] Corrao G, Corazza G, Bagnardi V, Brusco G, Ciacci C, Cottone M (2001). Mortality in patients with coeliac disease and their relatives: a cohort study. Lancet.

[B8] Veyrieres M, Baillet P, Hay JM, Fingerhut A, Bouillot JL, Julien M (1997). Factors influencing longterin survival in 100 cases of small intestine primary adenocarcinoma. Am J Surg.

[B9] Ludvigsson JF, Reutfors J, Osby U, Ekbom A, Montgomery SM Coeliac disease and risk of mood disorders – A general population-based cohort study. J Affect Disord.

[B10] Howdle PD, Holmes GK (2004). Small bowel malignancy in coeliac disease. Gut.

[B11] Kingham JG, Ramanaden D, Dawson A (1998). Metachronous small-bowel adenocarcinoma in celiac disease: gluten-free diet is not protective. Scand J Gastroenterol.

